# Universal membrane-labeling combined with expression of Katushka far-red fluorescent protein enables non-invasive dynamic and longitudinal quantitative 3D dual-color fluorescent imaging of multiple bacterial strains in mouse intestine

**DOI:** 10.1186/s12866-019-1538-z

**Published:** 2019-07-18

**Authors:** Oula Peñate-Medina, Robert J. Tower, Tuula Peñate-Medina, Olga Will, Per E. J. Saris, Juho Suojanen, Timo Sorsa, Laura Huuskonen, Kaisa Hiippala, Reetta Satokari, Claus C. Glüer, Willem M. de Vos, Justus Reunanen

**Affiliations:** 1 Molecular Imaging North Competence Center, Section Biomedical Imaging, Department of Radiology and Neuroradiology, University Hospital Schleswig-Holstein, Am Botanischen Garten 14, 24118 Kiel, Germany; 2 Department of Food and Environmental Sciences, University of Helsinki, Viikinkaari 9, 00014 Helsinki, Finland; 3 Cleft Palate and Craniofacial Centre, Department of Plastic Surgery, Helsinki University Hospital, Helsinki University Central Hospital, Topeliuksenkatu 5, 00029 Helsinki, Finland; 4 Päijät-Häme Joint Authority for Health and Wellbeing, Department of Oral and Maxillo-Facial Surgery, Keskussairaalankatu 7, 15850 Lahti, Finland; 5 Department of Oral and Maxillofacial Diseases, University of Helsinki and Helsinki University Hospital, Haartmaninkatu 4E, 00029 Helsinki, Finland; 6 Division of Periodontology, Department of Dental Medicine, Karolinska Institutet, Alfreds Nobels Alle 8, Huddinge, 14104 Stockholm, Sweden; 7 Department of Bacteriology and Immunology and Immunobiology Research Program, Faculty of Medicine, University of Helsinki, Haartmaninkatu 2, 00014 Helsinki, Finland; 8 Department of Veterinary Biosciences, University of Helsinki, Agnes Sjöberginkatu 2, 00014 Helsinki, Finland; 9 Laboratory of Microbiology, Wageningen University, Wageningen, 6708 PB The Netherlands; 10 Biocenter Oulu & Cancer and Translational Medicine Research Unit, University of Oulu, Aapistie 5, 90220 Oulu, Finland

**Keywords:** Bacterial colonization, Fluorescence imaging, Katushka, Dual-color imaging, Three-dimensional, Fluorescence molecular tomography, Murine model, Far-red fluorescent protein, In vivo, Intestine

## Abstract

**Background:**

The human gastrointestinal (GI) tract microbiota has been a subject of intense research throughout the 3rd Millennium. Now that a general picture about microbiota composition in health and disease is emerging, questions about factors determining development of microbiotas with specific community structures will be addressed. To this end, usage of murine models for colonization studies remains crucial. Optical in vivo imaging of either bioluminescent or fluorescent bacteria is the basis for non-invasive detection of intestinal colonization of bacteria. Although recent advances in in vivo fluorescence imaging have overcome many limitations encountered in bioluminescent imaging of intestinal bacteria, such as requirement for live cells, high signal attenuation and 2D imaging, the method is still restricted to bacteria for which molecular cloning tools are available*.*

**Results:**

Here, we present usage of a lipophilic fluorescent dye together with Katushka far-red fluorescent protein to establish a dual-color in vivo imaging system to monitor GI transit of different bacterial strains, suitable also for strains resistant to genetic labeling. Using this system, we were able to distinguish two different *E. coli* strains simultaneously and show their unique transit patterns. Combined with fluorescence molecular tomography, these distinct strains could be spatially and temporally resolved and quantified in 3D.

**Conclusions:**

Developed novel method for labeling microbes and identify their passage both temporally and spatially in vivo makes now possible to monitor all culturable bacterial strains, also those that are resistant to conventional genetic labeling.

**Electronic supplementary material:**

The online version of this article (10.1186/s12866-019-1538-z) contains supplementary material, which is available to authorized users.

## Background

A wealth of knowledge about microbiota composition in the gastrointestinal tract (GI-tract) has been gained from large international efforts such as the MetaHIT and the Human Microbiome Project [1]. These studies have revealed the microbial world within each of us to be composed of hundreds of species, with the whole microbial catalogue of the human GI-tract being made up thousands of bacterial species [2], out of which more than 1000 species have been cultured [3]. Our microbiota serves us in many ways, e.g. by fermenting nutrients otherwise undigestible, by synthesizing vitamins, and by out-competing pathogens [4, 5]. Reduction in diversity and changes in microbiota composition have been linked to GI-tract illnesses, such as ulcerative colitis and Crohn’s disease [6, 7]. Moreover, distinctive microbiota patterns have also been associated with diet-induced systemic disorders, such as obesity [8–10], metabolic syndrome [11, 12], type II diabetes [13] and cardiovascular disease [14], indicating the importance of GI-tract function in systemic health. According to the current scheme, the resilience provided by a “healthy” microbiota can be gradually deteriorated by external disturbances such as a high-fat diet or antibiotic use, which, in the onset of malignant processes, perturbs the GI-tract ecosystem leading to reduced microbiota diversity, low grade inflammation, and ultimately chronic disease development [5].

The concept of enterotypes was recently introduced by Arugaman et al., who used a combinatorial approach to analyze metagenomic data sets of 39 individuals from 4 countries in 3 different continents [15]. According to the authors, there are three robust bacterial clusters, the enterotypes, found in the GI-tract microbiomes across the human population, which are independent of the nation or continent of origin. Each of these enterotypes are built around stable bacterial communities composed of a limited number of species, suggesting that variations in microbiota structures between individuals are stratified rather than continuous. An important consequence of this observation is that there are a limited number of different stable host-microbial species combinations, and therefore a limited amount of symbiotic or mutualistic host-microbiota states that could differentially respond to external stimuli such as diet or medication. Based on metagenomic data sets and solid patterns of co-existing and negatively-correlating microbial genera (e.g. *Akkermansia* and *Ruminococci*), the enterotypes are assumed to be built upon trophic metabolic chains [15]. A choice between different enterotypes seems to be environmentally and microbially driven rather than determined by host genetics, since the core forming genera of each different enterotypes were found in all human subjects analyzed [16]. Furthermore, in a long-term follow-up study of more than 10 years, it was observed that individual’s microbiota can switch enterotype over time [17]. These findings, combined with data from patients treated for recurrent *Clostridium difficile* colitis [18] and insulin insensitivity [19] by fecal transplantation, support the emerging concept of personalized microbiota modulation as a future therapy to treat multiple disorders [20].

**Fig. 1:**
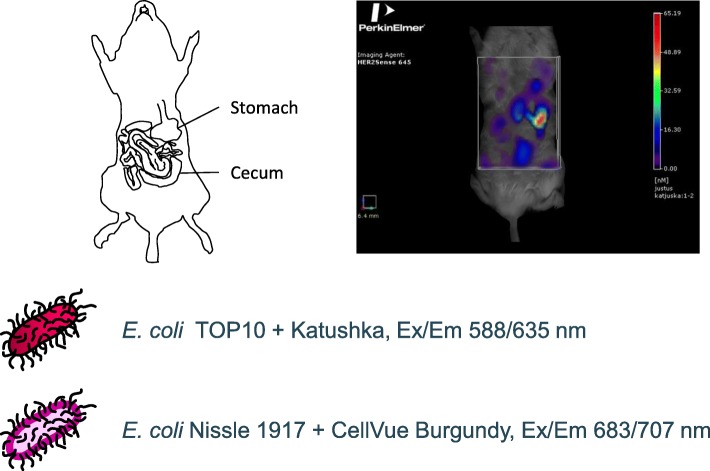
Non-invasive 3D dual-color imaging of fluorescent bacteria in murine intestine. 3D imaging combined with 2D imaging and post mortem histopathology allows assessment of the dynamic relationships between different bacterial populations and their locations in organ, tissue and cell level. 2D imaging and histopathology can be used to calibrate and locate the tissues in dynamic real-time settings


Despite the increasing body of evidence from human cohorts linking microbiota aberrations with major human diseases, most of the functional proof-of-concepts on the role of microbiota and its individual members in health and disease have been obtained from mouse intervention studies using gnotobiotic (germ-free) mice [21]. The gnotobiotic mouse model continues to be instrumental in the study of the microbiota’s effects on host physiology and health, since it offers a reductionistic (both in terms of microbiota and host genetics) in vivo model, in which the effects of pre-designed minimal microbiota, composed of a single or a few species only, can be monitored. To date, the monitoring of bacterial species and strains in the mouse GI-tract has been based on either in vivo bioluminescence or fluorescence of genetically labeled strains*,* in vitro analysis of fecal samples, or ex vivo analysis of resected intestinal tissue. All these approaches are subject to several drawbacks: i) bioluminescent imaging of bacteria in the GI tract has been limited to 2-dimensional imaging and thus poor spatial resolution [22–24], ii) high tissue absorption of fluorescent signal at wavelengths below the far-red area of the visible spectrum iii) fluorescence imaging with near-infrared fluorescent proteins requires addition of biliverdin, an exogenous chromophore needed for fluorescence development [25] iv) fecal samples reflect colonic microbiota only, whereas microbiota composition and function differ markedly between the small intestine and colon [19, 26–28], v) resected tissues represent end-point analysis only, which disregards dynamic fluctuations in host-microbe and microbe-microbe relationships over time [29]. The dual-color fluorescent labeling method presented herein, using either genetically encoded Katushka far-red fluorescent protein or membrane-labeling fluorescent dye for strains not amenable for expression of foreign proteins, combined with fluorescence molecular tomography (FMT) (Fig. 1; Additional file 1: Movie S1), overcomes many of these limitations by offering the possibility to do simultaneous 3D imaging of different microbes in mouse intestine for non-invasive, real-time, in vivo analysis of host-microbe and microbe-microbe interplay.

**Additional file 1: Movie S1.** 3D video of mouse fed with fluorescent bacteria. (WMV 651 kb)


## Results

### Movement of bacteria along the GI tract can be monitored in 3D using fluorescently-labeled bacteria

**Fig. 2:**
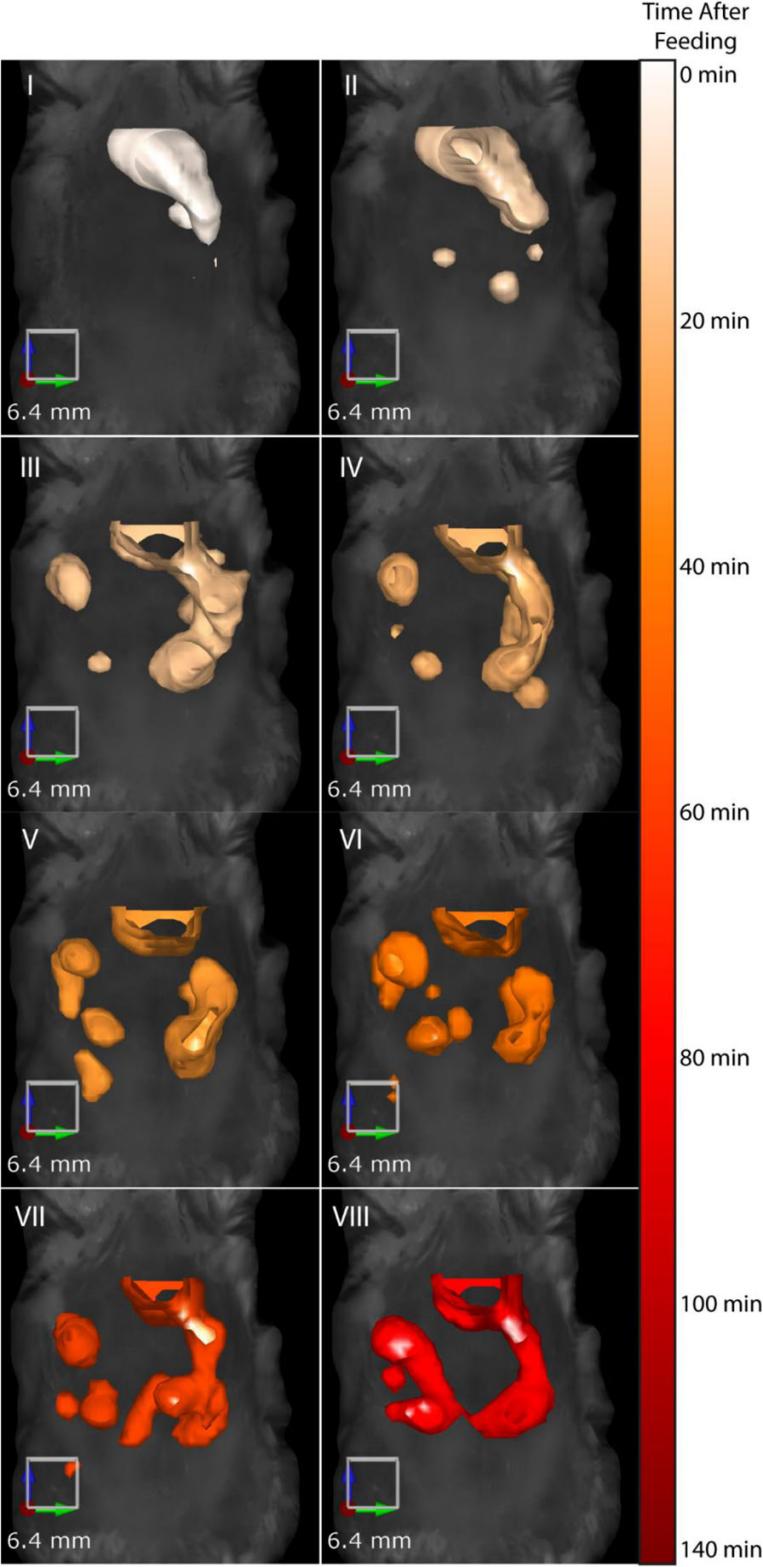
Fluorescently-labeled *E. coli* Nissle 1917 can be tracked in vivo over time after feeding. Mice were fed fluorescently-labeled *E. coli* Nissle 1917 and imaged over time by FMT. Images were acquired at 10, 30, 45, 60, 80, 95, 110 and 120 min after feeding and show isosurface rendering of fluorescent signal

**Fig. 3:**
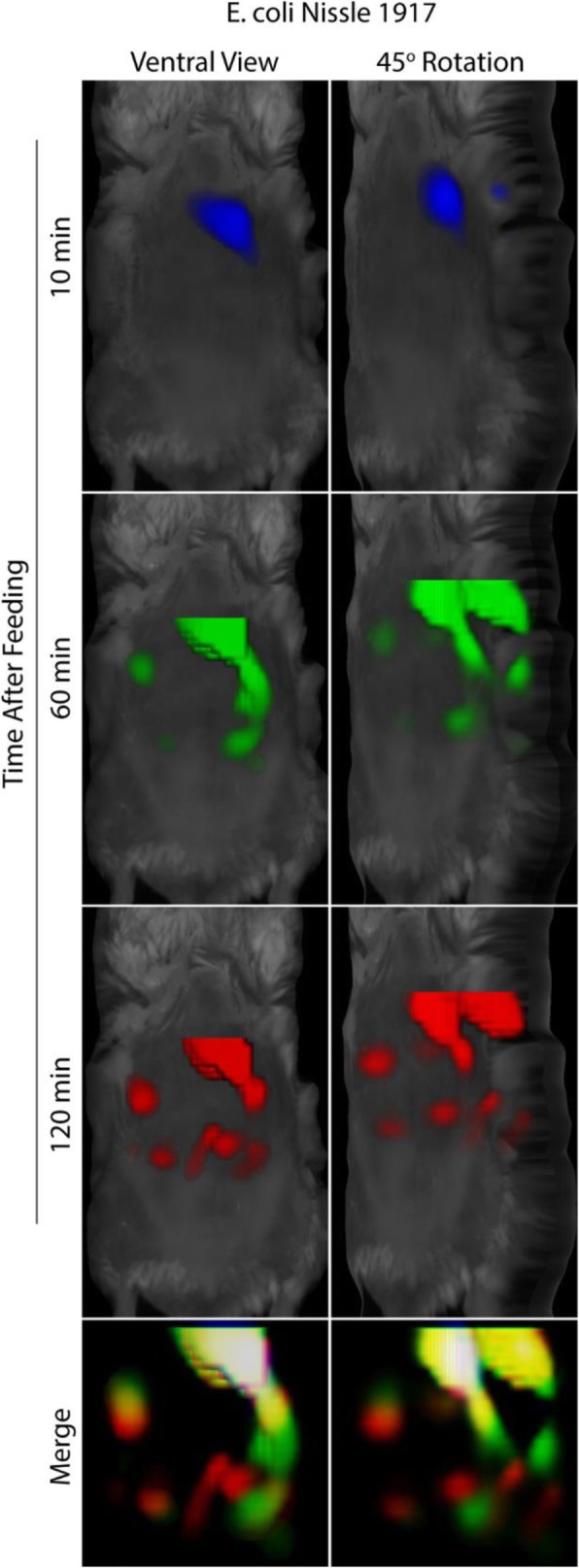
In vivo imaging using FMT allows for temporal and 3D spatial monitoring of bacterial progression. Mice fed with fluorescent *E. coli* Nissle 1917 were imaged over time without repositioning. Reconstructed images were merged allowing both temporal and 3D spatial monitoring of bacteria progression

*E. coli* Nissle 1917 bacteria were labeled with a fluorescent dye, fed to mice, and monitored over time using in vivo FMT (Fig. 2). Images show the bacteria progressing through the small intestine. FMT also permits the generation of temporal 3D images of bacterial localization as shown by FMT images of mice imaged without repositioning in between scans. Overlaying images from different time points reveals initial stomach emptying and 3D localization of bacteria over time (Fig. 3).

### Dual-color FMT imaging reveals the differences in intestinal localizations of labeled bacterial strains

**Fig. 4:**
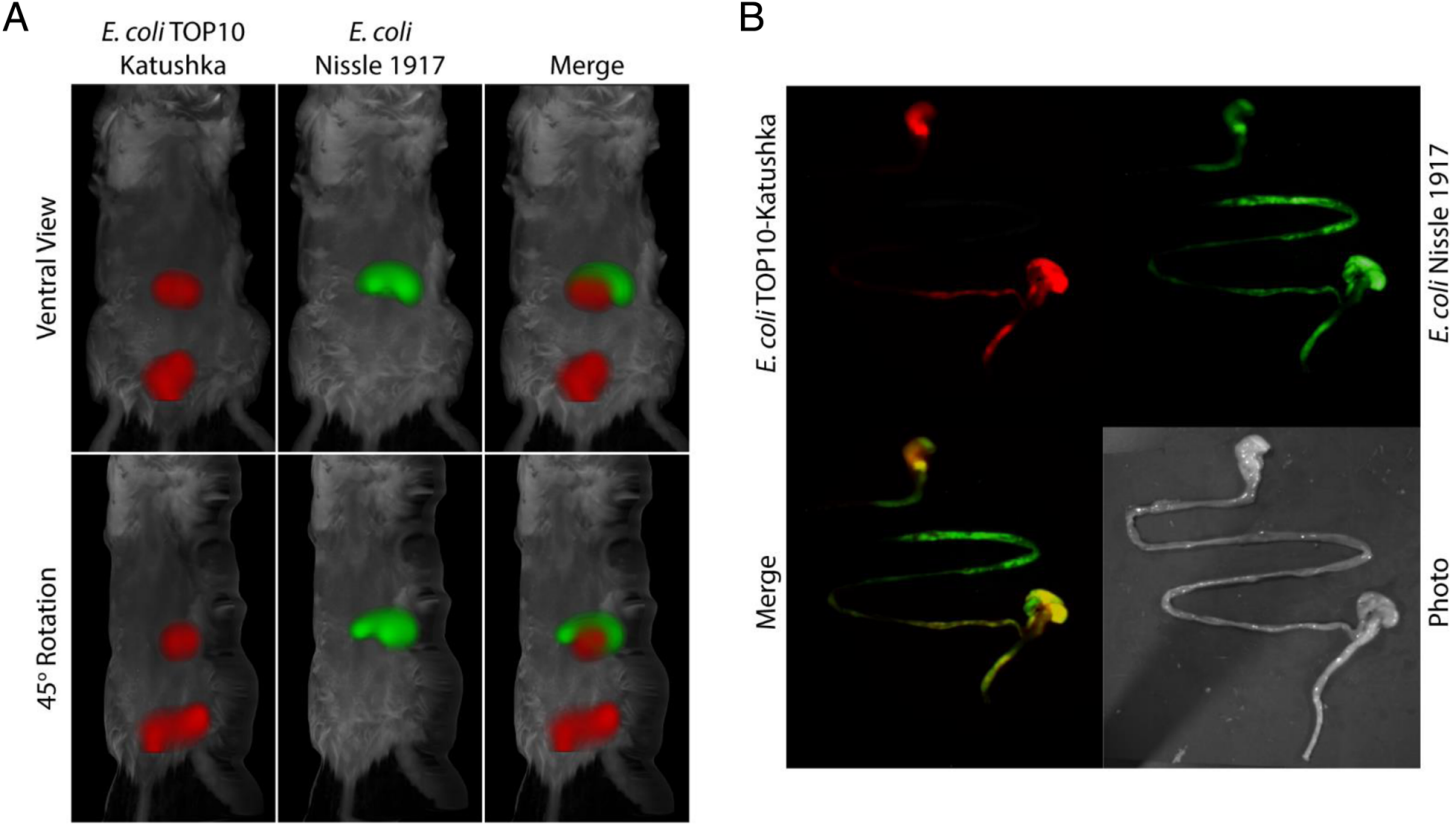
Mice co-fed with colonizing and non-colonizing bacteria show differential localization 24 h after feeding. Mice were co-fed colonizing, fluorescently-labeled *E. coli* Nissle 1917 and the TOP10 bacteria constitutively expressing the fluorescent protein Katushka. 24 h after feeding, FMT images show a distinct localization pattern with the Nissle 1917 bacteria found within the upper digestive tract while the TOP10 bacteria are present much lower within the mice (**a**). Ex vivo analyses of the intestinal tract by NightOwl imaging show substantial amounts of Nissle 1917 cells present in the early ileum while the TOP10 bacteria are more confined to the terminal ileum and colon (**b**)

The *E.coli* K12-derived TOP10 bacteria, stably expressing the near far-red fluorescent protein Katushka, and the fluorescently-labeled *E. coli* Nissle 1917 were co-fed to mice and imaged 24 h after feeding to determine their differential localization within the digestive tract (Fig. 4a). Merged images show that, while Nissle 1917 is predominantly located in the upper digestive tract, the TOP10 bacteria are present primarily at the lower part of the intestine. Regions of fluorescence were confirmed by ex vivo planar fluorescent imaging of the intestinal tract (Fig. 4b). Ex vivo analyses of the excised digestive tract showed Nissle 1917 bacteria in the duodenum and jejunum/early ileum while the TOP10 bacteria were primarily located in the terminal ileum and colon.

### FMT imaging allows quantitative monitoring of spatially-resolved bacteria in the GI tract

**Fig. 5:**
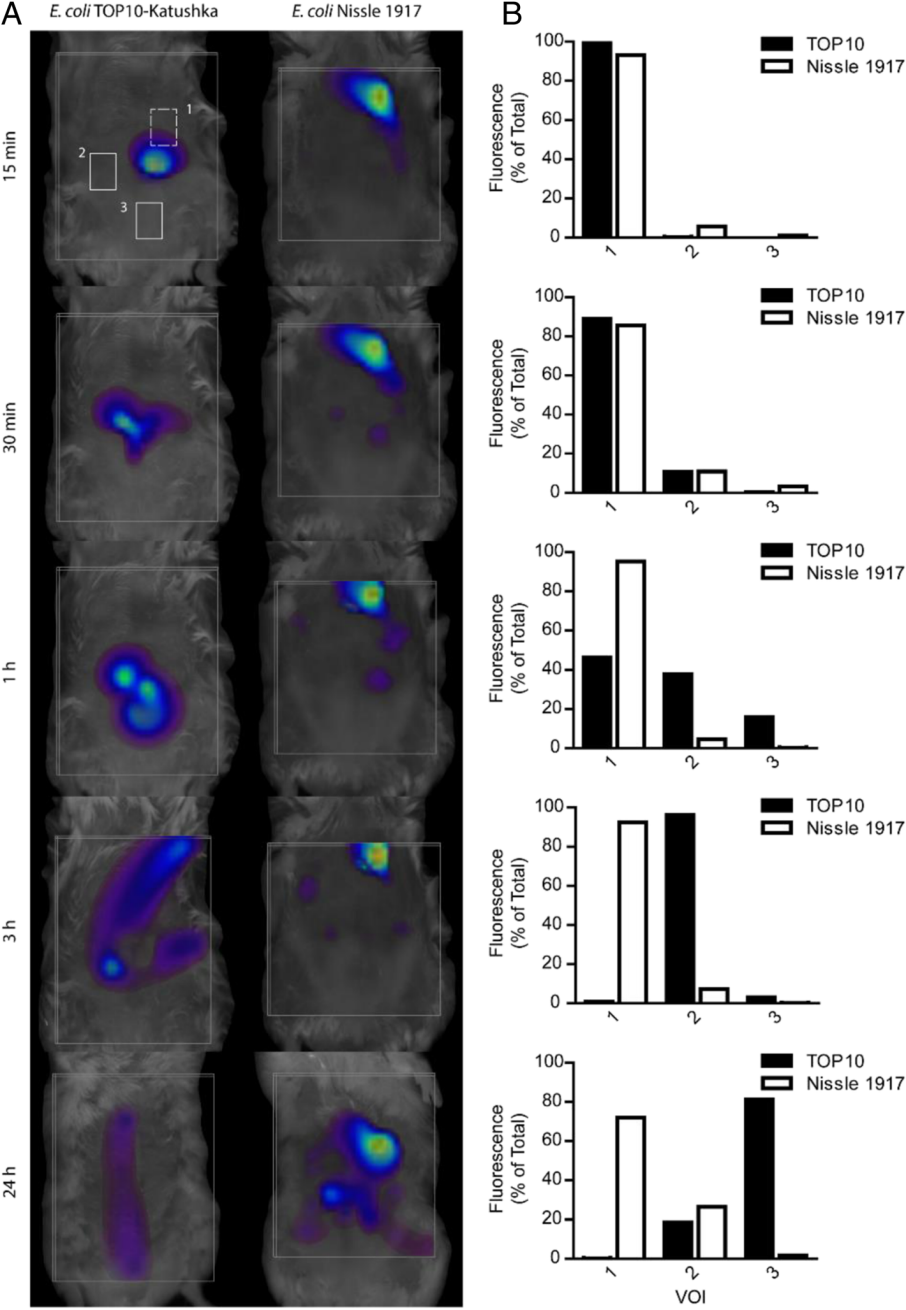
3D in vivo imaging shows increased retention of colonizing bacteria in the early digestive tract compared with non-colonizing bacteria. Mice fed with fluorescently-labeled Nissle 1917 or Katushka-expressing TOP10 were imaged by FMT over time to track the progression of the bacteria (**a**). VOIs approximating the stomach through upper small intestine (1), lower small intestine (2), and cecum/colon (3) were placed on 3D reconstructed images. Dashed boxes represent VOIs placed towards the dorsal side of the mouse while solid boxes represent regions in the ventral side. (**b**) Fluorescences of individual VOIs were quantified and distribution of each bacteria over the 3 selected regions were determined. The Nissle 1917 shows preferential retention in regions 1 and 2 over the 24 h period while the TOP10 progresses through the digestive tract with time and is primarily found in regions 2 and 3 after 24 h

Movement of bacteria along the GI tract was analyzed by placing volumes of interest (VOIs) on mice fed with fluorescent bacteria and monitored in vivo over time (Fig. 5a). VOIs were quantified at each time point to assess *E. coli* TOP10 and Nissle 1917 strains progression through the GI-tract (Fig. 5b). Twenty-four hours after feeding, a substantial retention of Nissle 1917 in the upper and lower small intestine (VOIs 1 and 2, respectively) was observed, whereas the majority of the TOP10-derived fluorescence was found in the area spanning the terminal ileum (VOI 2), and cecum/colon (VOI 3). The weakest fluorescent foci observed by FMT for *E. coli* Nissle 1917, stained with a lipophilic membrane dye, and TOP10, expressing the Katushka fluorescent protein, corresponded to approximately 3 × 10^5^ and 1 × 10^6^ colony forming units (cfu), respectively.

## Discussion

Here we present data on a fluorescence-based dual-color imaging system for bacteria in mouse intestine. In this system, one bacterial strain was metabolically labeled with the near far-red fluorescent protein Katushka [30], while the second bacterial strain was surface-labeled with an infrared red membrane-dye. The usage of fluorescent reporters in the study of microbial ecology in the intestine has been hampered by the autofluorescent and light absorbing properties of several tissues, e.g. the high absorbance by hemoglobin in the visible spectrum (< 650 nm) and by lipids and water in the infrared range (> 900 nm). The detrimental effect of tissue autofluorescence in intestinal whole body imaging is exemplified by the usage of low wavelength green fluorescent protein (GFP) as a reporter, where at least 10^11^ colony forming units (cfu) were required to obtain bacterial GFP signal in vivo from mouse intestine [31]. Given that the physiological levels of major species in the colon are in the range of 10^9^–10^10^ cfu/g digesta, development of more sensitive reporter systems has been warranted. Tissue absorbance and autofluorescence (which are substantially diminished at higher wavelengths), combined with light refractive properties governing tissue penetration, create an optimal biological imaging window in the red/infrared area of the spectrum, where in vivo*,* whole-body, light-based imaging is maximized. Attempts to answer this demand have included introduction of different red-fluorescent proteins from multiple sources, such as mCherry and DsRed [32, 33], but the relative fluorescence intensities of these proteins have been rather disappointing in in vivo imaging applications. These sensitivity-related issues were overcome by the introduction of bioluminescent labeling of bacteria for whole animal intestinal imaging in 2006 [34], when Wiles et al. transformed the enteric mouse pathogen *Citrobacter rodentium* with a plasmid harboring the luxCDABE gene cassette [35]. This bioluminescence-based in vivo imaging strategy was sensitive enough to detect *lux*-labeled bacteria in the murine intestine after oral cavage with 10^9^ cfu bacteria, which is in the range of densities determined for single bacterial species in the mammal intestine, and has been the preferred choice for in vivo imaging of intestinal microbes ever since. There are, however, several important limitations in the usage of bioluminescence: the resolution of luminescence imaging does not allow visualization of individual bioluminescent bacterial cells. Furthermore, even if detection of single cells was not desired, bioluminescence is dependent on cellular NAPDH and can therefore only be used to detect metabolically active bacteria, thus excluding the possibility for most ex vivo microscopical analyses of bioluminescently-labeled bacteria in tissue samples. Most importantly, although bioluminescent reporter systems with different light spectra have been described [23, 36], the spatial information of murine gut colonization using bioluminescent dual-color imaging remains scarce, since according to our knowledge no bioluminescent dual-color imaging system suitable for 3D imaging of GI-tract bacteria has been reported to date. In contrast, current advances in fluorescence imaging of GI-tract bacteria have addressed many of these shortages, e.g. simultaneous dual-color 3D imaging of different bacteria utilizing expression of spectrally different infrared fluorescent proteins has been presented recently [25]. However, usage of infrared fluorescent proteins not only requires addition of exogenous biliverdin as a co-factor for fluorophore maturation and thus development of fluorescence, but is also limited to bacterial strains for which molecular cloning tools are available.

Here, we seek to overcome the present shortcomings encountered with bioluminescent labeling and earlier fluorescent approaches by combining metabolic labeling with the far-red fluorescent protein Katushka with a membrane-labeling IR dye, thus combining genetic labeling with universal membrane-labeling suitable for all cultivable bacteria. We chose the K12-derivative TOP10 as a representative of commensal *E. coli*, and Nissle 1917 strain for its well characterized probiotic properties. The strain K12 belongs to the *E. coli* reference strain collection [37] major phylogenetic lineage A, that comprises mostly commensal isolates, whereas Nissle 1917 strain falls into the phylogroup B2 containing many of the extraintestinal pathogenic *E. coli* (ExPEC) strains [38]. A common feature of representatives of the B2 group is the prevalence of certain virulence-associated genes (e.g. fimbriae, capsular antigens, α-hemolysin, and colibactin), and it has been noted that accumulation of these pathogenicity determinants correlated positively with intestinal residency time of commensal isolates [38]. Interestingly, it has been shown that the presence of the colibactin-encoding *pks*-island associates with long-term colonization as compared with intermediate-term or transiently colonizing *E. coli* isolates [39]. Furthermore, the ability of the Nissle 1917 strain to exert its probiotic effects cannot be separated from its capability to produce active colibactin, blurring the thin lines between probiocity, commensalism, and pathogenicity [40].

The excitation and emission characteristics of Katushka (588 nm_ex_, 635 nm_em_) allow it to be used for dual-color imaging in combination with several dyes fluorescing in the infrared region. In whole body imaging, the near far-red and IR fluorescent regions are superior over lower wavelengths due to minimal tissue autofluorescence and light absorption at these wavelengths. Here we show that FMT imaging of live mice fed with fluorescently-labeled bacteria can be used for tracking the bacteria over time in 3D, and for identifying differentially moving bacterial foci with 3D image overlays. We additionally show, that mice fed simultaneously with two different *E. coli* strains, Nissle 1917 and TOP10, show discrete localizations and colonization time and patterns within the GI tract and can easily be distinguished using fluorescence imaging. And finally, the bacterial movement through the GI-tract can be quantified showing a more rapid progression for K12-derived TOP10 bacteria and prolonged retention for Nissle 1917 strain known to possess high capacity for long-term colonization of the gut [41]. Total GI fluorescence was used to quantify the minimum intensity foci observed in fed mice and was found to correlate with ~ 3 × 10^5^ cfu. This estimation reflects the smallest observed colonization site and does not necessarily reflect the absolute lowest detection limit of FMT. It is interesting to note that fluorescence at intensities of 100 fold lower values than those reported in this study are capable of detection by FMT. It is also important to note that estimations derived here make the false assumption that the entire bacterial load fed to the mice survive passaging through the stomach and enter then GI-tract. These facts suggest our estimated 3 × 10^5^ cfu threshold could reflect a significant overestimation. Sensitivity will also be affected by the tissue depth from which the signal originates, as well as the wavelength of fluorescent molecule used, with longer wavelengths being associated with more efficient tissue penetration.

## Conclusion

The 3D fluorescent imaging method for bacteria in mouse intestine presented here should find wide usage in the study of many aspects of intestinal microbial ecology. Being quantitative and dual-colored, it allows simultaneous monitoring and enumeration of different bacterial strains in the distinct topological parts of the intestine, and by being non-invasive and real-time, it substantially reduces the number of animals needed to achieve this goal. Most importantly, even though the repertoire of cultivable human gut microbiota members exceeds 1000 nowadays, most of them still remain non-transformable, thus necessitating usage of non-genetic labeling methods. As such, the method described herein represents a major development paving the way for increasing our knowledge of the highly complex host-microbe and microbe-microbe interactions taking place inside the dynamic milieu of the mammal intestine.

## Methods

### Bacterial strains and labeling

Synthetic DNA containing Katushka fluorescent protein encoding gene under the control of the LeuS-promoter was ordered from GeneArt (ThermoFisher Scientific). This DNA fragment was cloned into the plasmid pLEB124 using standard molecular cloning protocols. The ligation product was transformed into the K12-derived *E. coli* strain TOP10 (Invitrogen) to produce a Katushka-expressing, near far-red fluorescing TOP10 strain. *E. coli* Nissle 1917 cells were labeled with the CellVue Burgundy Cell labeling kit (Ebioscience, Frankfurt, Germany). For mouse experiments the bacteria were grown overnight at 37 °C on LB-agar (Nissle 1917) or on LB-agar supplemented with 200 μg/ml erythromycin (Katushka-expressing TOP10), and washed once with phosphate buffered saline (PBS) prior to feeding to the mice.

### Animal experiments

10 week old C57BL/6 albino mice (in mixed sex relationships) were used from in-house breeding. The breeding animals were acquired at Charles Rives Laboratory (Sulzfeld, Germany). All animals were kept in a temperature and humidity-controlled environment, with a 12 h light/dark cycle, with access to food and water ad libitum. Animal experiments and care were in accordance with the guidelines of institutional authorities and approved by the Ethics Committee for Animal Experiments at Christian-Albrechts-Universität-zu-Kiel [approval number 312–7224.121-17 (46–3/13)]. For imaging experiments, the mice (*n* = three per group) were fed 5 × 10^7^ cfu *E. coli* cells suspended into 50 μl PBS. Mice were anesthetized with intraperitoneal injections of 80 mg/kg ketamine (Aveco Pharmaceutical, IA) and 0.5 mg/kg dorbene (Pfizer, Berlin, Germany). For long-term anesthetization, additional half-dose administrations of ketamine and dorbene were given upon initial signs of waking. At the end of the experiment, all mice were killed by overdose of anesthesia.

### Imaging

In vivo imaging was done using fluorescence molecular tomography on a FMT2500LX (Perkin Elmer, MA). Reconstructed images were analyzed and volumes of interest (VOIs) placed using the TrueQuant software. Ex vivo imaging was done using the NightOwl planar imaging system and Indigo software (Berthold Technologies, Bad Wildbad, Germany). Merged images were prepared using ImageJ. Calibration of the FMT for lipophilic-stained and Katushka-expressing bacterial cells was determined by averaging the total fluorescence of the GI tract from all images taken within 2 h of feeding (before any significant clearance of the bacteria could occur) and setting that equal to the initial bacterial feeding (5 × 10^7^ cfu). Sensitivity was assessed by quantifying the foci with the minimal fluorescent intensity.

## Data Availability

All data generated or analysed during this study are included in this published article and its supplementary information file.

## Abbreviations

cfu: colony forming unit
ExPEC: Extraintestinal pathogenic *E. coli*
GI: Gastrointestinal
PBS: Phosphate buffered saline
VOI: Volume of interest
